# Reduction of liquid terminated-carboxyl fluoroelastomers using NaBH_4_/SmCl_3_

**DOI:** 10.1039/c9ra10069e

**Published:** 2020-03-17

**Authors:** Yunfei Chang, Mingyi Liao, Xueyan Li

**Affiliations:** College of Transportation Engineering, Dalian Maritime University No. 1 Linghai Road Dalian 116026 China liaomy2000@sohu.com

## Abstract

Using a simple one-pot method, the reduction of liquid terminated-carboxyl fluoroelastomers (LTCFs) by sodium borohydride and samarium chloride (NaBH_4_/SmCl_3_) was successfully realized and liquid terminated-hydroxyl fluoroelastomers (LTHFs) were obtained. The structure and functional group content of LTCFs and LTHFs were analyzed by FTIR, ^1^H-NMR, ^19^F-NMR and chemical titration. The results showed that –C=C– and carboxyl groups of LTCFs were reduced efficiently, the reduction rate reached 92% under optimum reaction conditions. Compared with other frequently-used metal chlorides, SmCl_3_ with a high coordination number could increase the reduction activity of NaBH_4_ more effectively and the reduction mechanism was explored.

## Abstract 2

A facile method using NaBH_4_/SmCl_3_ allows for reduction of liquid terminated-carboxyl fluoroelastomers (LTCFs) in excellent yields and provides an attractive potential scheme for the reduction of other carboxyl organic compounds.

## Graphical abstract

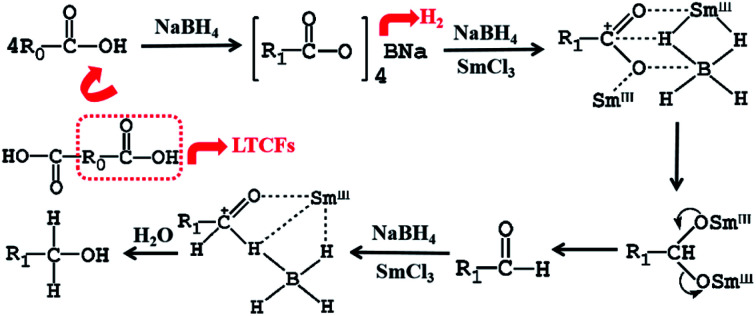

## 1. Introduction

As an important polymer material, fluoroelastomers have been widely utilized in the field of chemical engineering, machinery and aerospace^1–3^ due to their inherent resistance to fuel, oil, high temperature and oxidation. The stabilized carbon–fluorine single bond (C–F 485 kJ mol^−1^) and the shielded effect of fluorine atoms on the main chain^4,5^ make the chemical properties of fluoroelastomers more stable than other rubbers. Curable liquid fluoroelastomers with lower molecular weight have attracted much attention using solvent-free sealant and adhesive formulators because of their liquidity and plasticity. Low molecular weight LTCFs with *M*_n_ ranging from 500 to 10 000 were prepared by oxidative degradation of fluoroelastomers.^6,7^ However, due to the properties of carboxyl groups, the LTCFs are difficult to react with cross-linking agents at low temperature and have low heat resistance stability,^8^ which will inevitably affect their comprehensive properties and limit their application fields. Therefore, it is particularly important to convert the carboxyl groups into hydroxyl groups to improve the thermal stability and reduce the curing temperature of liquid fluoroelastomers.

While significant advances had been made in reduction reactions including for carbonyl compounds, these processes often did not reduce carboxyl groups directly.^9–12^ Because in contrast to ketones,^13^ aldehydes^14^ and esters,^15^ which easily engage in reactions with reductive agents, carboxylic acids are generally unreactive.^16^ On the other hand, although lithium aluminium hydride was widely used as a strong reductant in carboxyl reduction,^17^ its poor chemical selectivity, flammability and explosive nature are unfavorable for large-scale production. Kamochi and Kudo reported the reduction of aryl carboxylic acid derivatives and some aliphatic carboxylic acids using SmI_2_,^18,19^ however this strategy was low yielding and limited in scope for aliphatic carboxylic acids. Szostak *et al.*^20^ developed a method for efficient electron transfer reduction of carboxylic acids using SmI_2_–H_2_O–Et_3_N and achieved excellent yield, the protocol was not suitable for large-scale production because SmI_2_ was costly and difficult to store.

NaBH_4_ has attracted much attention due to its applications in reduction in organic chemistry. Compared with lithium aluminium hydride and SmI_2_, NaBH_4_, with moderate cost, mild reaction conditions and excellent chemical selectivity, is conducive to safe production. Nonetheless, NaBH_4_ does not directly reduce the carboxylic acids by reason of its relatively weak reductive activity. To reduce carboxylic acid compounds effectively, many Lewis acids have been developed to improve the reduction activity of NaBH_4_ including I_2_,^21^ ZnCl_2_,^22^ LiCl,^23^ AlCl_3_,^24^ CoCl_2_ (ref. 25) and so on. Although the LTCFs were reduced by NaBH_4_/I_2_, the addition of I_2_ complicated the post-treatment process and the residual iodine would severely affect the stability of products.^26^ Hence, further systematical studies on the reduction of LTCFs are academically and industrially significant. Rare earth ions with strong positive electricity, high coordination number and outer empty orbit have strong oxygen affinity.^27^ Therefore, they can complex with ethers, carbonyl compounds, nitrogen compounds and so on.^28^ The unique electronic and chemical properties of rare earth ions make them have many advantages, for instance, mild reaction conditions, satisfactory selectivity, less environmental pollution and high efficiency catalytic cycles.^29^ Hence, our interest in a new strategy for reductive transformations by using NaBH_4_ and rare earth ions led us to introduce NaBH_4_/SmCl_3_ as the first reagent to selectively reduce LTCFs. This mainly involves a simple one-pot process with mild reaction conditions and the NaBH_4_/SmCl_3_ dosage, reaction temperature, reaction time and organic solvents were systematically studied. To the best of our knowledge, few facile methods have been reported to date for the reduction of carboxylic acids by NaBH_4_/SmCl_3_.

## 2. Experimental section

### 2.1 Chemicals

Vinylidene fluoride-hexafluoropropylene copolymer (VDF-HFP) with VDF/HFP = 3/1 and *M*_n_ = 78 000 was purchased from Chenguang Research Institute of Chemical Industry (China). Hydrogen peroxide (H_2_O_2_), potassium biphthalate (C_8_H_5_KO_4_), phenolphthalein, bromothymol blue, potassium hydroxide (KOH), acetone and benzyltriethylammonium chloride (BTEAC) were purchased from Alfa Aesar (China). NaBH_4_, AlCl_3_, CoCl_2_, ZnCl_2_, CaCl_2_ and SmCl_3_ were purchased from Aladdin Industrial Corporation (Shanghai, China). Tetrahydrofuran (THF), 0.1 mol L^−1^ hydrochloric acid (HCl) and diglyme were purchased from Kermel Reagent (Tianjin, China).

### 2.2 Synthesis of LTCFs^9^

LTCFs were prepared through chemically degradation of high molecular weight solid VDF-HFP copolymer. High molecular weight VDF-HFP (100 g) and acetone (300 mL) were added into a 2000 mL flask. The mixture was stirred at room temperature for 24 h till the copolymer was completely dissolved. Under stirring at 0 °C, BTEAC (6.5 g), 30 wt% H_2_O_2_ aqueous solution (60 g), 45 wt% KOH aqueous solution (43 g) were added sequently. The reaction mixture was then warmed up to 24 °C and stirred for 7 h. After the reaction, the organic and inorganic phases were separated by filtration. HCl was used to acidify the organic phases and then the mixture was washed by excess deionized water for 3 times. Finally, the deposition was dried under 60 °C for 48 h in a vacuum drying chamber to obtain LTCFs.

### 2.3 Synthesis of LTHFs

5.000 g LTCFs (2.66% carboxyl content) were dissolved in a mixture of THF (15 mL) and diglyme (15 mL) for 1 h, then 0.447 g NaBH_4_ was dissolved into the solution under stirring for 1 h at 0 °C. Subsequently, 1.506 g SmCl_3_ was added into the system and temperature rose to 90 °C. After 6 h, the inorganic impurities were dissolved by 30 mL 0.1 mol L^−1^ HCl and the mixture was washed by excess deionized water for 3 times. Finally, the deposition was dried under 60 °C for 48 h in a vacuum drying chamber to obtain LTHFs.

### 2.4 Characterization

ATR-FTIR measurement was performed using a PerkinElmer Instruments Spectrum One. FTIR spectra were obtained at the resolution 4 cm^−1^ in the range 650–4000 cm^−1^. The NMR spectra were analyzed by Bruker AC 80 spectrometers (500 MHz for ^1^H, 470 MHz for ^19^F) at room temperature, using acetone-d_6_ as the solvent and TMS as internal standard the references for ^1^H (or ^19^F) nuclei. Chemical shifts are reported in ppm.

### 2.5 Chemical titration

#### 2.5.1 Preparation and calibration of KOH/C_2_H_5_OH solution

0.1 mol L^−1^ KOH/C_2_H_5_OH solution was prepared in volumetric bottle according to the China National Standards GB/T 603-2002 and GB/T 601-2002. 0.2 g of potassium biphthalate and 40 μL phenolphthalein solution (10 g L^−1^) was dissolved in water (50 mL). KOH/C_2_H_5_OH solution was added dropwise into the potassium biphthalate and phenolphthalein aqueous solution and when the aqueous solution changed from colorless to pink, it was the end point of titration. Blank experiment as the comparison was done. The concentration of KOH/C_2_H_5_OH standard titration solution was calculated by formula (1):1
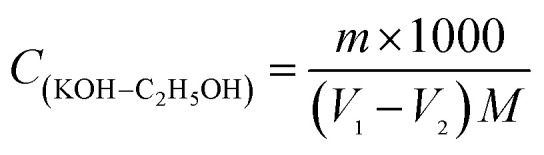
*V*_1_: volume of KOH/C_2_H_5_OH solution consumed by the potassium biphthalate and phenolphthalein solution (mL), *V*_2_: volume of KOH/C_2_H_5_OH solution consumed by blank sample (mL), *m*: weight of potassium biphthalate (g), *M*: 204.22 g mol^−1^.

#### 2.5.2 Determination of carboxyl content

0.75 g of LTCFs and 40 μL bromothymol blue (10 g L^−1^) was dissolved in acetone (40 mL). KOH/C_2_H_5_OH solution was used for titration and the LTCFs and bromothymol blue solution changed from light yellow to green, which was the end point of titration. Carboxyl content of LTCFs was calculated by formula (2):2
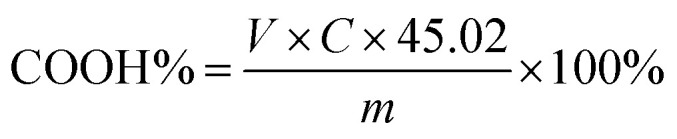
*V*: volume of KOH/C_2_H_5_OH solution consumed by the LTCFs and bromothymol blue solution (mL), *C*: the concentration of KOH/C_2_H_5_OH standard titration solution (mol L^−1^), *m*: weight of LTCFs (g).

#### 2.5.3 Determination of hydroxyl content

Reductive rate of hydroxyl was calculated by the LTCFs and LTHFs content of carboxyl (3).3
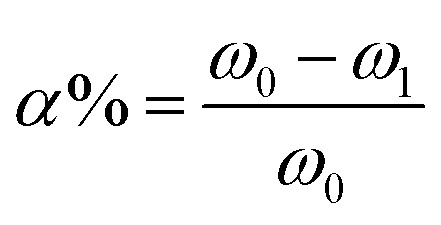
where *ω*_0_: LTCFs content of carboxyl, *ω*_1_: LTHFs content of carboxyl.

## 3. Results and discussion

### 3.1 Effect of reaction conditions

**Fig. 1:**
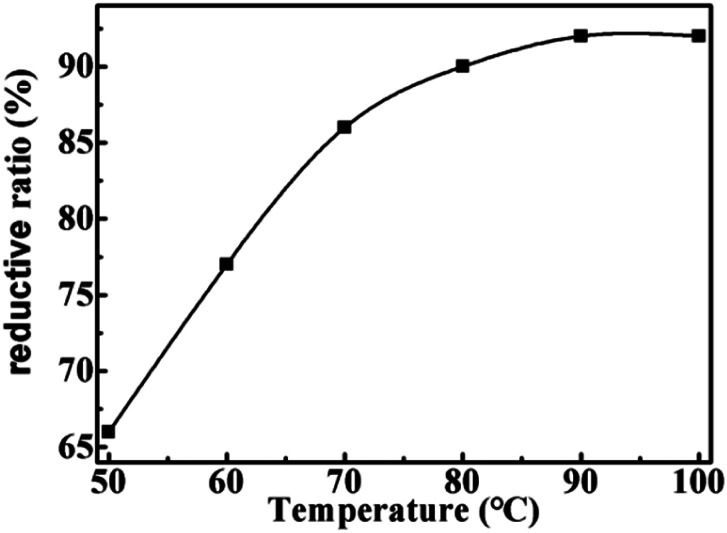
The effect of temperature on the reductive rate of LTCFs.

**Fig. 2:**
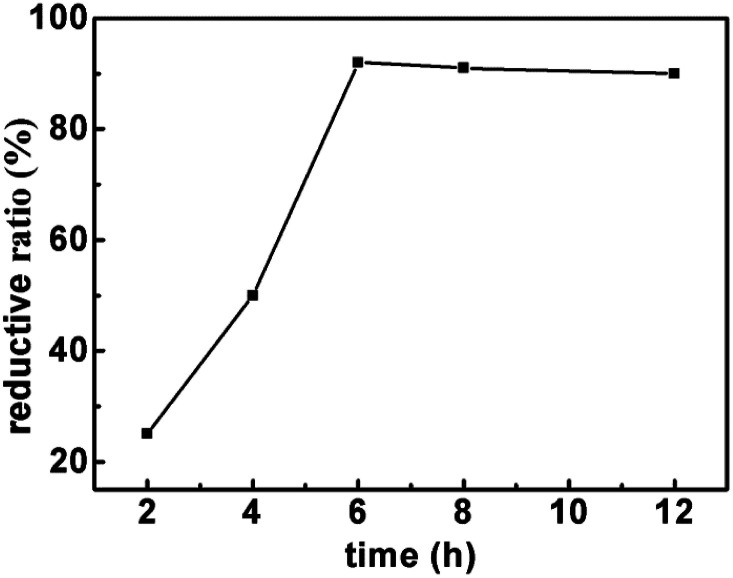
The effect of time on the reductive rate of LTCFs.

Firstly, the effect of temperature on reductive rate is examined. As shown in Fig. 1, the reduction rate of LTCFs increases with the increasing temperature and reaches the maximum at 90 °C. Secondly, the effect of reaction time is also investigated and the results are shown in Fig. 2, the reductive rate of LTCFs increases with increasing reaction time. When reaction time is 6 h, the reductive rate reaches the maximum. It indicates that the optimum reaction temperature is 90 °C and optimum reaction time is 6 h.

### 3.2 Effect of reduction system proportion

**Fig. 3:**
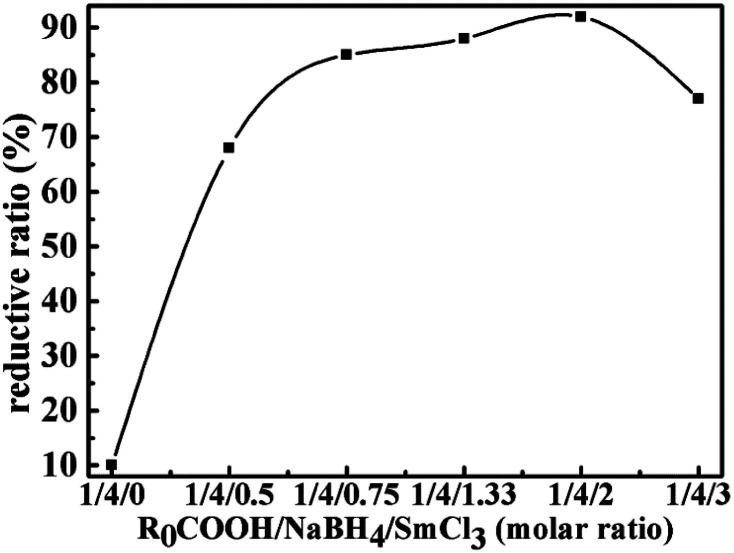
The effect of amount of SmCl_3_ on the reductive rate of LTHFs.

Table 1 shows the effect of NaBH_4_/SmCl_3_ system and solvents on the reduction reaction under the optimum reaction temperature and time. We find that on the basis of the chemical titration, the NaBH_4_/SmCl_3_ system in THF/diglyme could reduce LTCFs efficiently. As shown in No. 1 and 2 of Table 1, the necessity of each component of the reduction system is investigated. No reductive reaction is observed without NaBH_4_ and only 12% reduction rate is obtained at the absence of SmCl_3_, which indicates that SmCl_3_ has no reducibility to LTCFs and NaBH_4_ only has little reducibility. However, the reducibility increases significantly when the reductive system is composed of the two above reagents. The reductive rate of LTCFs increases with increasing the NaBH_4_/SmCl_3_ dosage and the optimal molar ratio of R_0_COOH/NaBH_4_/SmCl_3_ is 1/4/2 (No. 3–6). When molar ratio of R_0_COOH/NaBH_4_/SmCl_3_ further increases to 1/5/2.5, the reduction rate is decreasing due to the coating of the NaBH_4_ particles with the excess SmCl_3_, which likely impedes the efficient solubility of NaBH_4_ (No. 7). Furthermore, as shown in Fig. 3, the SmCl_3_ addition amount is investigated. When the molar ratio of NaBH_4_/SmCl_3_ changes from 4/0 to 4/2, the reductive rate of LTCFs increases with increasing amount of SmCl_3_. Nevertheless, when the molar ratio is 4/3, the excess SmCl_3_ make the reductive rate decrease because the coating of the NaBH_4_ particles with the excess SmCl_3_.

**Table 1:** The effect of NaBH_4_/SmCl_3_ system on the reduction of LTCFs

No.	R_0_COOH	NaBH_4_	SmCl_3_	THF/diglyme (mL)	Reductive rate (%)
1	1	0	3	1/1 (30 mL)	0
2	1	6	0	1/1 (30 mL)	12
3	1	1	0.5	1/1 (30 mL)	13
4	1	2	1	1/1 (30 mL)	48
5	1	3	1.5	1/1 (30 mL)	61
6	1	4	2	1/1 (30 mL)	92
7	1	5	2.5	1/1 (30 mL)	88
8	1	4	2	1/0 (30 mL)	80
9	1	4	2	2/1 (30 mL)	85
10	1	4	2	1/2 (30 mL)	86
11	1	4	2	1/1 (20 mL)	53
12	1	4	2	1/1 (40 mL)	59
13	1	4	2	1/1 (50 mL)	42

### 3.3 Effect of solvent on reductive rate

As shown in Table 1 from No. 8, although the NaBH_4_/SmCl_3_ system in THF readily reduces LTCFs to their corresponding LTHFs, it has a lower reduction rate. The low reduction rate is possibly due to the weak of solubility of NaBH_4_ in THF. To improve the solubility of NaBH_4_ in THF, varying amounts of diglyme, a known excellent solvent for NaBH_4_, are added. After some optimizations, the 1/1 volume ratio of THF/diglyme mixed solvent is adequate to solubilize NaBH_4_ (No. 6, 8–10). In addition, the effect of mixed solvent amount on reduction rate is also investigated (No. 6, 11, 12, 13). The results show that the maximum reduction rate is obtained as the amount of mixed solvent is 30 mL. The lower solvent amount will lead to the decrease solubility of NaBH_4_ and the excessive use will affect the complexation of NaBH_4_/SmCl_3_ with carboxyl group. Consequently, 30 mL of THF/diglyme (1/1 volume ratio) mixed solvent is selected to investigate the reduction of LTCFs.

From above experimental results we can conclude that the optimal reaction conditions are as follows: the reaction temperature is 90 °C, the reaction time is 6 h, the molar ratio of the R_0_COOH/NaBH_4_/SmCl_3_ is 1/4/2 and the THF/diglyme volume ratio is 1/1. Under the optimal reaction conditions the LTCFs are converted to LTHFs in a reductive rate about 92%. For the scale-up, when we expand the experiment by 30 times, the reduction rate do not decrease significantly. Therefore, the method can be expected in industrial utility.

As comparison, several other metal chlorides (MCl_*x*_) are also evaluated. Through the optimization studies, the optimal reaction conditions of NaBH_4_/MCl_*x*_ are consistent with NaBH_4_/SmCl_3._ As shown in Table 2, only NaBH_4_/SmCl_3_ gives excellent reductive rate of LTCFs (No. 1). It is concluded that the unique electronic structure and high coordination number of samarium ions provide a new method for efficient reduction of LTCFs and a potential scheme for the reduction of other carboxyl organic compounds.

**Table 2:** Comparison of reduction activity between NaBH_4_/MCl_*x*_ and NaBH_4_/SmCl_3_

Reduction system	Ratio (mol)	THF/diglyme (mL)	Reductive rate (%)
NaBH_4_/SmCl_3_	4/2	1/1	92
NaBH_4_/AlCl_3_	4/2	1/1	64
NaBH_4_/ZnCl_2_	4/2	1/1	72
NaBH_4_/CaCl_2_	4/2	1/1	44
NaBH_4_/CoCl_2_	4/2	1/1	20

### 3.4 Structure characterization

**Fig. 4:**
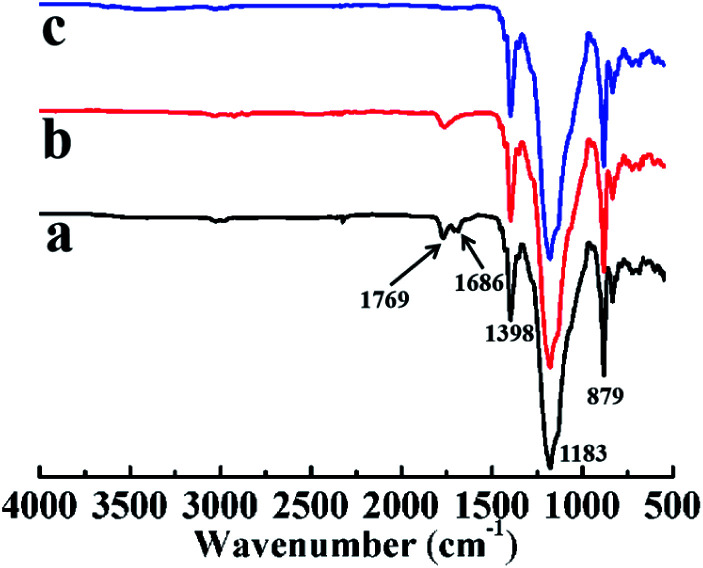
FTIR spectra of (a) LTCFs, (b) reduction product of single NaBH_4_ (No. 2) and (c) LTHFs (No. 6).

FTIR spectra of LTCFs is shown in Fig. 4(a). It can be seen from Fig. 4(a) that the absorption peaks at 1769, 1686, 1398, 1183, and 879 cm^−1^ ascribed to stretching vibration of –CF_2_COOH, –C=C–, –FCH_2_–, –CF_2_–, and –CF_3_, respectively.^30,31^Fig. 4(b) shows the FTIR spectra of reduction product by single NaBH_4_. Comparing the Fig. 4(b) with Fig. 4(a), the absorption peak of –C=C– at 1686 cm^−1^ is weakened and the absorption peak of –CF_2_COOH is still obvious (1769 cm^−1^) indicating that the structure of –C=C– is reduced significantly but only little –CF_2_COOH is reduced *via* single NaBH_4_ without SmCl_3_. Fig. 4(c) shows the FTIR spectra of LTHFs reduced by NaBH_4_/SmCl_3_. From Fig. 4(c) can be seen that the absorption peaks of –C=C– and –CF_2_COOH are weakened ascribed to effective reduction of NaBH_4_/SmCl_3_. LTHFs exhibits absorption peaks at 1398, 1183 and 879 cm^−1^ ascribed to stretching vibration of –FCH_2_–, –CF_2_–, and –CF_3_, respectively, which clearly shows that LTHFs have the same backbone structure as LTCFs.

**Fig. 5:**
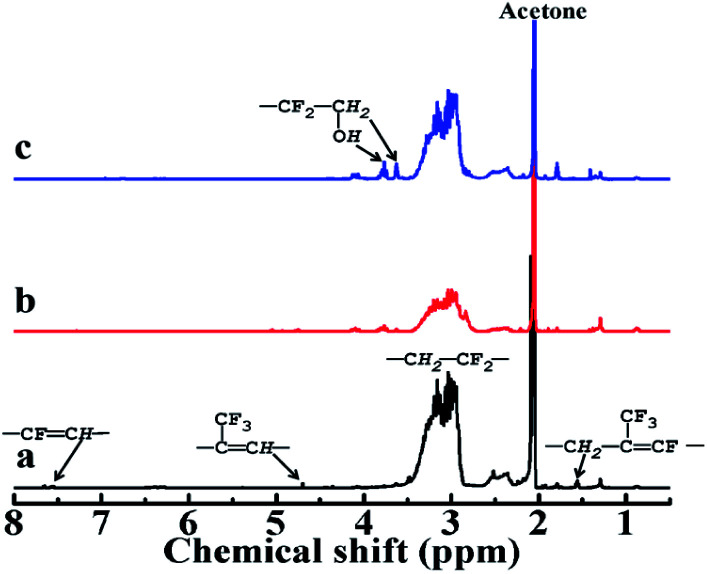
^1^H-NMR spectra of (a) LTCFs, (b) reduction product of single NaBH_4_ reductant (No. 2) and (c) LTHFs (No. 6).

As seen from Fig. 5(a), in the ^1^H-NMR spectra of LTCFs the multiple peaks at 3.51–2.86 ppm, the peaks at 1.55 ppm, 4.68 ppm and 7.50–7.70 ppm are assigned to the structures of –CH_2_CF_2_–, –CF=C(CF_3_)CH_2_–, –(CF_3_)C=CH– and –CH=CF–,^32,33^ Respectively. Fig. 5(b) shows the disappearance of –C=C– peaks at 1.55 ppm, 4.68 ppm and 7.5–7.70 ppm, respectively, indicating that –C=C– converted to –C–C–. In general, single NaBH_4_ does not reduce –C=C– and it is commonly necessary to add Lewis acids to improve the reduction activity.^34,35^ However, –C=C– of LTCFs can be directly reduced by single NaBH_4_ ascribed to effect of F atom, which increases the activity of –C=C– through the electronic induction effect. Compared with the LTCFs, as seen from Fig. 5(c), LTHFs exhibit new peaks at 3.63 ppm and 3.75 ppm, which are assigned to the structure of –CH_2_OH,^30^ it clearly confirms the formation of hydroxyl groups.

**Fig. 6:**
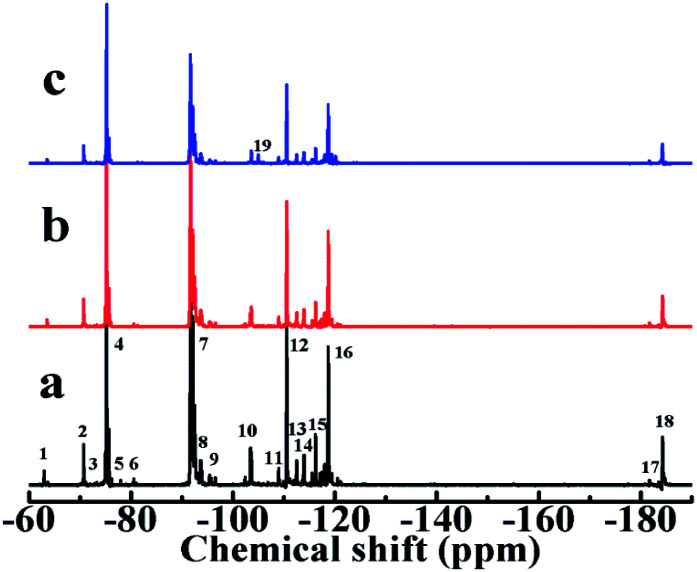
^19^F-NMR spectra of (a) LTCFs, (b) reduction product of single NaBH_4_ reductant (No. 2) and (c) LTHFs (No. 6).

**Fig. 7:**
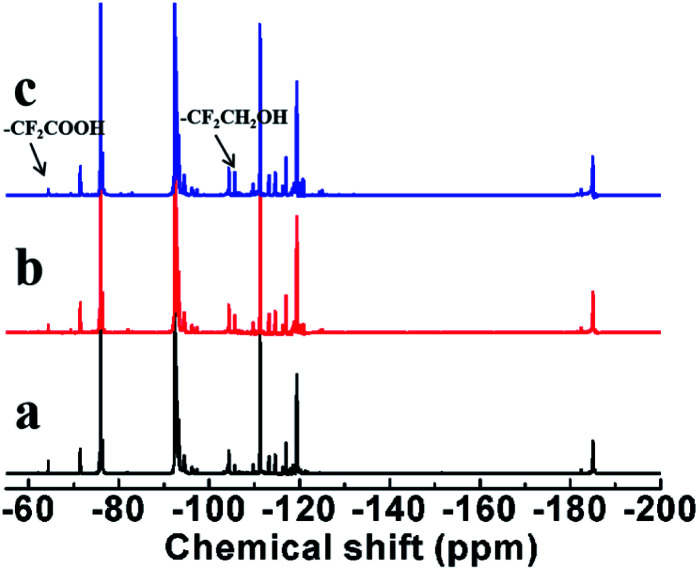
^19^F-NMR spectra of LTHFs in different reaction times ((a) 1 h, (b) 3 h, (c) 5 h).

^19^F-NMR spectra were used further to characterize. ^19^F-NMR spectra of (a) LTCFs, (b) reduction product of single NaBH_4_ reductant (No. 2) and (c) LTHFs (No. 6) are shown in Fig. 6. The assignments of peaks are listed in Table 3. As seen from Fig. 6(a), a peak at 63.67 ppm is ascribed to –CF_2_COOH. The peaks at −73.71 ppm, 80.66 ppm and 81.30 ppm are ascribed to the fluorine atoms on the structure of –(CF_3_)C=CH–, –CH=CF– and –CF=C(CF_3_)CH_2_– for LTCFs^36–38^ respectively. Fig. 6(b) shows that –C=C– peaks at −73.71 ppm, 80.66 ppm and 81.30 ppm disappear by reductive transformation using NaBH_4_, this result is similar to Fig. 5(b). Fig. 6(c) clearly shows new peak at −104.9 ppm, which is assigned to –CF_2_CH_2_OH.^30^ Therefore, the above results of ^19^F-NMR spectra, ^1^H-NMR and FTIR spectra consistent with each other and LTHFs is prepared successfully. ^19^F-NMR spectra of LTHFs in different reaction times is shown in Fig. 7. It can be seen from Fig. 7 that no byproducts are detected in all times.

**Table 3:** Assignments of ^19^F-NMR peaks in liquid fluoroelastomers

No	*δ* (ppm)	Assignment	No	*δ* (ppm)	Assignment
1	−63.46	–CF_2_CF_2_COOH	11	−108.96	–CF(CF_3_)CH_2_CF_2_CF(CF_3_)CF_2_–
2	−70.67	–CH_2_CF_2_CF(CF_3_)CF_2_CH_2_–	12	−110.51	–CF_2_CH_2_CF_2_CF_2_CF(CF_3_)–
3	−73.71	–CF_2_CH=C(CF_3_)CF_2_–	13	−112.53	–CF(CF_3_)CH_2_CF_2_CF_2_CH_2_–
4	−75.19	–CF_2_CH_2_CF(CF_3_)CF_2_CF_2_–	14	−113.95	–CF_2_CH_2_CF_2_CF_2_CH_2_–
5	−80.66	–CH=CFCF(CF_3_)–	15	−116.24	–CH_2_CH_2_CF_2_CF_2_CF(CF_3_)–
6	−81.30	–CF=CHCF(CF_3_)CF_2_–	16	−118.72	–CH_2_CF_2_CF_2_CF(CF_3_)CH_2_–
7	−91.62	–CF_2_CH_2_CF_2_CH_2_CF_2_–	17	−181.74	–CH_2_CF_2_CF(CF_3_)CF_2_CH_2_–
8	−93.56	–CF_2_CH_2_CF_2_CH_2_CF(CF_3_)–	18	−184.33	–CF_2_CF_2_CF(CF_3_)CH_2_CF_2_–
9	−95.64	–CH_2_CH_2_CF_2_CH_2_CF_2_–	19	−104.90	–CF_2_CH_2_OH
10	−103.62	–CF_2_CH_2_CF_2_CF(CF_3_)CF_2_–			

### 3.5 Reduction mechanism

**Scheme 1:**
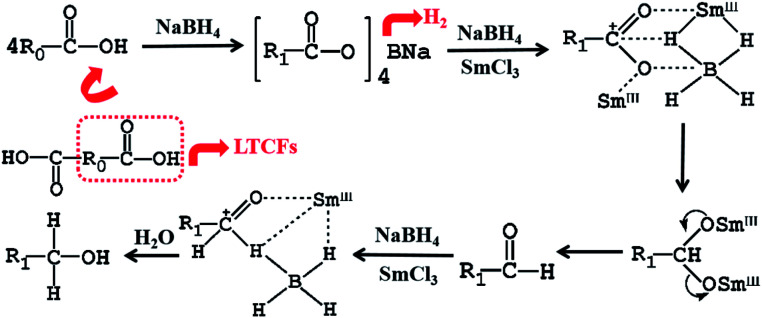
Scheme of reduction mechanism of LTCFs (R_0_ is composed of –CH_2_CF_2_–, –CF_2_CF(CF_3_)–, –CFCHCHF(CF_3_)–, –CF_2_C(CF_3_)CHCF_2_–, and –CFCH–; R_1_ is composed of –CH_2_CF_2_– and –CF_2_CF(CF_3_)–).

The reported literatures on reduction mechanism of NaBH_4_ and Lewis acid has predominantly considered that borane (BH_3_) was formed by the reaction of NaBH_4_ with Lewis acid initially, and then BH_3_ reduced the substrates.^21,39^ To investigate the applicability of this reduction mechanism in our experiment for LTCFs, the sequence of reagent addition is investigated first (Table 4). When NaBH_4_, SmCl_3_ and LTCFs are added into the reactor simultaneously, the reduction rate of LTCFs is 33% (No. 1). When SmCl_3_ and LTCFs are first added into the reactor under stirring for 1 h, then, NaBH_4_ is added and the reduction rate is 48% (No. 2). The above two addition sequence experiments can ensure the reaction of NaBH_4_ with SmCl_3_ firstly and have low reduction rate indicating the mechanism is not suitable for LTCFs. Nevertheless, when NaBH_4_ and LTCFs are first added into the reactor under stirring for 1 h, then SmCl_3_ is added (No. 3), the reduction rate increase dramatically to 92%. Hence, according to our experimental results and literatures,^20,40^ the reduction mechanism of LTCFs is proposed, as shown in Scheme 1. Firstly, –C=C– of LTCFs is directly reduced by NaBH_4_ and R_0_COOH react with NaBH_4_ to form (R_1_COO)_4_BNa and release H_2_. Secondly, the complexation of Sm^3+^ with carbonyl oxygen of LTCFs makes the electrons of carbonyl group move to oxygen and increase the electrophilicity of carbonyl carbon. Electrophilicity of carbonyl carbon is facilely attacked by the borohydride anion (BH_4_^−^) of NaBH_4_.^40^ Thirdly, with the departure of Sm^3+^ and the other oxygen atom, aldehyde group is formed.^20^ Aldehyde group is then reduced by NaBH_4_/SmCl_3_ and the LTCFs is successfully reduced to the final formation of LTHFs. In contrast to carboxyl group, aldehyde group is readily reactive. The generated aldehyde group in the reaction could be immediately reduced by the strong reduction system of NaBH_4_/SmCl_3_. Therefore, no byproduct of liquid terminated-aldehyde fluoroelastomers is generated.

**Table 4:** Effect of charging sequence on the reduction of LTCFs^a^

No.	Step 1a	Step 2b	Reductive rate (%)
1	NaBH_4_, SmCl_3_, LTCFs	—	33
2	SmCl_3_, LTCFs	NaBH_4_	48
3	NaBH_4_, LTCFs	SmCl_3_	92

a First, chemical reagent of Step 1a were added into the reactor and reacted 1 h. Then, chemical reagent of Step 2b was added for reaction.

## 4. Conclusion

In summary, the combination of affordable and readily available NaBH_4_/SmCl_3_ provided a method for the selective reduction of LTCFs in THF/diglyme and the 1/4/2 molar ratio of R_0_COOH/NaBH_4_/SmCl_3_ was suitable for LTCFs reduction at 90 °C. LTCFs were reduced to their corresponding LTHFs in excellent reduction rate (92%). FTIR spectra and NMR spectra analysis showed that –C=C– and –CF_2_COOH of LTCFs were effectively reduced to –C–C– and –CF_2_CH_2_OH. We have proposed the reduction mechanism that Sm^3+^ complex with the carbonyl group increase the electroaffinity of the carbon of carbonyl and make carbon of carbonyl more receptive to the hydride moiety transfer from the borohydride anion. Application of this method to reduction of other carboxylic acid derivatives will be presented in our future work.

## Conflicts of interest

The authors declare no competing financial interests.

## Supplementary Material
